# Supplementary material for: Water production efficiency and economic benefits under diversified planting modes of intercropping-multiple cropping in arid regions
Source: PLoS One. 2025 Mar 10;20(3):e0318540. doi: 10.1371/journal.pone.0318540 (PMC11892835; doi:10.1371/journal.pone.0318540)
Supplement: Attachment — Submitted filename: PONE-D-24-46367.pdf [file pone.0318540.s002.pdf]

PLOS ONE

Water production efficiency and economic benefits under diversified planting modes of intercropping-multiple cropping in arid regions

--Manuscript Draft--

**Manuscript Number:**	PONE-D-24-46367
**Article Type:**	Research Article
**Full Title:**	Water production efficiency and economic benefits under diversified planting modes of intercropping-multiple cropping in arid regions
**Short Title:**	Research on Diversified Planting Modes
**Corresponding Author:**	Na Zhang<br>Ningxia Hui Autonomous Region Water Resources Science & Research Institute<br>yinchuan, China, CHINA
**Keywords:**	intercropping-multiple cropping; diversified planting; land equivalent ratio; economic performance; arid area
**Abstract:**	Diversified planting patterns are important measures to improve the comprehensive grain production capacity, alleviate the contradiction between grain crops and forage planting, and enhance water production efficiency. In order to explore the suitable diversified planting modes in the Yellow River irrigation area of Ningxia, a total of 4 diversified planting modes were designed, including intercropping wheat with maize silage and replanting sorghum-sudangrass hybrid after wheat harvest (T1), intercropping wheat with cabbage, replanting oil sunflower after wheat harvest, and replanting maize silage after cabbage harvest (T2), single wheat, after wheat harvest, half of the land is replanted with maize silage, and half of the land is replanted with oil sunflower (T3), and single maize silage (T4). The results showed that all diversified planting modes can increase biomass and land equivalent ratio compared to the control monoculture. The T2 had the highest biomass, with an average two-year increase of 103.01% compared to the T4, and an average land equivalent ratio of 2.27. After wheat harvesting, the highest biomass yield per cubic meter of water was achieved by T3, with an average of 12.65 kg/m3 over two years. The highest with an average two-year benefit per cubic meter of water was achieved by T1, with an average of 16.81 Chinese Yuan/m3. When two crops are intercropped, maize silage is at a competitive advantage in both T1 and T2 treatments, with a relative competitive ability between 0.059-0.234. When maize silage and oil sunflower are planted simultaneously, oil sunflower is the dominant crop. The net benefit of T1 treatment is the highest, with an average total net income of 85,000 Chinese Yuan/667m2 over two years. It can be seen that diversified planting patterns can improve the yield and economic benefits per unit land area, and are a highly promising planting pattern.
**Order of Authors:**	Na Zhang
	Jianxin Jin
	Jing Chen
**Additional Information:**	
**Question**	**Response**
**Financial Disclosure**<br><br>Enter a financial disclosure statement that describes the sources of funding for the work included in this submission. Review the submission guidelines for detailed requirements. View published research articles from *PLOS ONE* for specific examples.	Yes

Powered by Editorial Manager® and ProduXion Manager® from Aries Systems Corporation

This statement is required for submission and **will appear in the published article** if the submission is accepted. Please make sure it is accurate.<br><br>**Funded studies**<br>Enter a statement with the following details:<br>• Initials of the authors who received each award<br>• Grant numbers awarded to each author<br>• The full name of each funder<br>• URL of each funder website<br>• Did the sponsors or funders play any role in the study design, data collection and analysis, decision to publish, or preparation of the manuscript?<br><br>Did you receive funding for this work?	
Please add funding details.<br>as follow-up to "**Financial Disclosure**<br><br>Enter a financial disclosure statement that describes the sources of funding for the work included in this submission. Review the submission guidelines for detailed requirements. View published research articles from *PLOS ONE* for specific examples.<br><br>This statement is required for submission and **will appear in the published article** if the submission is accepted. Please make sure it is accurate.<br><br>**Funded studies**<br>Enter a statement with the following details:<br>• Initials of the authors who received each award<br>• Grant numbers awarded to each author<br>• The full name of each funder<br>• URL of each funder website<br>• Did the sponsors or funders play any role in the study design, data collection and analysis, decision to publish, or preparation of the manuscript?	This work was supported by the National Key Research & Development Program of China (2021YFD1900600, 2022YFG1900205); the Key Research & Development Program of Ningxia Hui Autonomous Region (2021BEF02034); the Ningxia Natural Science Foundation Project (2022AAC03725)

Did you receive funding for this work?"	
Please select the country of your main research funder (please select carefully as in some cases this is used in fee calculation).<br>as follow-up to "**Financial Disclosure**<br><br>Enter a financial disclosure statement that describes the sources of funding for the work included in this submission. Review the submission guidelines for detailed requirements. View published research articles from *PLOS ONE* for specific examples.<br><br>This statement is required for submission and **will appear in the published article** if the submission is accepted. Please make sure it is accurate.<br><br>**Funded studies**<br>Enter a statement with the following details:<br>• Initials of the authors who received each award<br>• Grant numbers awarded to each author<br>• The full name of each funder<br>• URL of each funder website<br>• Did the sponsors or funders play any role in the study design, data collection and analysis, decision to publish, or preparation of the manuscript?<br><br>Did you receive funding for this work?"	CHINA - CN
**Competing Interests**<br><br>Use the instructions below to enter a competing interest statement for this submission. On behalf of all authors, disclose any competing interests that could be perceived to bias this work—acknowledging all financial support and any other relevant financial or non-financial competing interests.<br><br>This statement is required for submission and **will appear in the published article** if	The authors have declared that no competing interests exist.

the submission is accepted. Please make sure it is accurate and that any funding sources listed in your Funding Information later in the submission form are also declared in your Financial Disclosure statement.<br><br>View published research articles from *PLOS ONE* for specific examples.<br><br>**NO authors have competing interests**<br><br>Enter: *The authors have declared that no competing interests exist.*<br><br>**Authors with competing interests**<br><br>Enter competing interest details beginning with this statement:<br><br>*I have read the journal's policy and the authors of this manuscript have the following competing interests: [insert competing interests here]*<br><br>* typeset	
**Ethics Statement**<br><br>Enter an ethics statement for this submission. This statement is required if the study involved:<br><br>• Human participants<br>• Human specimens or tissue<br>• Vertebrate animals or cephalopods<br>• Vertebrate embryos or tissues<br>• Field research<br><br>Write "N/A" if the submission does not require an ethics statement.<br><br>General guidance is provided below. Consult the submission guidelines for detailed instructions. **Make sure that all information entered here is included in the Methods section of the manuscript.**	N/A

**Format for specific study types**<br><br>**Human Subject Research (involving human participants and/or tissue)**<br>• Give the name of the institutional review board or ethics committee that approved the study<br>• Include the approval number and/or a statement indicating approval of this research<br>• Indicate the form of consent obtained (written/oral) or the reason that consent was not obtained (e.g. the data were analyzed anonymously)<br><br>**Animal Research (involving vertebrate animals, embryos or tissues)**<br>• Provide the name of the Institutional Animal Care and Use Committee (IACUC) or other relevant ethics board that reviewed the study protocol, and indicate whether they approved this research or granted a formal waiver of ethical approval<br>• Include an approval number if one was obtained<br>• If the study involved *non-human primates*, add *additional details* about animal welfare and steps taken to ameliorate suffering<br>• If anesthesia, euthanasia, or any kind of animal sacrifice is part of the study, include briefly which substances and/or methods were applied<br><br>**Field Research**<br><br>Include the following details if this study involves the collection of plant, animal, or other materials from a natural setting:<br>• Field permit number<br>• Name of the institution or relevant body that granted permission	
**Data Availability**<br><br>Authors are required to make all data underlying the findings described fully available, without restriction, and from the time of publication. PLOS allows rare exceptions to address legal and ethical concerns. See the PLOS Data Policy and FAQ for detailed information.	Yes - all data are fully available without restriction

A Data Availability Statement describing where the data can be found is required at submission. Your answers to this question constitute the Data Availability Statement and **will be published in the article**, if accepted.<br><br>**Important:** Stating 'data available on request from the author' is not sufficient. If your data are only available upon request, select 'No' for the first question and explain your exceptional situation in the text box.<br><br>Do the authors confirm that all data underlying the findings described in their manuscript are fully available without restriction?	
**Describe where the data may be found in full sentences. If you are copying our sample text, replace any instances of XXX with the appropriate details.**<br><br>• If the data are **held or will be held in a public repository**, include URLs, accession numbers or DOIs. If this information will only be available after acceptance, indicate this by ticking the box below. For example: *All XXX files are available from the XXX database (accession number(s) XXX, XXX.)*.<br>• If the data are all contained **within the manuscript and/or Supporting Information files**, enter the following: *All relevant data are within the manuscript and its Supporting Information files.*<br>• If neither of these applies but you are able to provide **details of access elsewhere**, with or without limitations, please do so. For example:<br><br>*Data cannot be shared publicly because of [XXX]. Data are available from the XXX Institutional Data Access / Ethics Committee (contact via XXX) for researchers who meet the criteria for access to confidential data.*<br><br>*The data underlying the results presented in the study are available from (include the name of the third party*	All relevant data are within the manuscript and its Supporting Information files.

and contact information or URL).<br>• This text is appropriate if the data are owned by a third party and authors do not have permission to share the data.<br><br>* typeset	
Additional data availability information:	Tick here if your circumstances are not covered by the questions above and you need the journal's help to make your data available.

Manuscript Click here to access/download;Manuscript;Main document.docx

Water production efficiency and economic benefits under diversified planting modes of intercropping-multiple cropping in arid regions

Na Zhang[1,2], Jianxin Jin[3], Jing Chen[2*]

1. Ningxia Hui Autonomous Region Water Conservancy Research Institute, Yinchuan 750021,China

2. Hohai University, Nanjing 211100, China

3. Institute of Agricultural Resources and Environment, Ningxia Academy of Agricultural and Forestry Sciences, Yinchuan 750002, China

* Correspondence: 2156640980@qq.com

Abstract: Diversified planting patterns are important measures to improve the comprehensive grain production capacity, alleviate the contradiction between grain crops and forage planting, and enhance water production efficiency. In order to explore the suitable diversified planting modes in the Yellow River irrigation area of Ningxia, a total of 4 diversified planting modes were designed, including intercropping wheat with maize silage and replanting sorghum-sudangrass hybrid after wheat harvest (T1), intercropping wheat with cabbage, replanting oil sunflower after wheat harvest, and replanting maize silage after cabbage harvest (T2), single wheat, after wheat harvest, half of the land is replanted with maize silage, and half of the land is replanted with oil sunflower (T3), and single maize silage (T4). The results showed that all diversified planting modes can increase biomass and land equivalent ratio compared to the control monoculture. The T2 had the highest biomass, with an average two-year increase of 103.01% compared to the T4, and an average land equivalent ratio of 2.27. After wheat harvesting, the highest biomass yield per cubic meter of water was achieved by T3, with an average of 12.65 kg/m^3 over two years. The highest with an average two-year benefit per cubic meter of water was achieved by T1, with an average of 16.81 Chinese Yuan/m^3. When two crops are intercropped, maize silage is at a competitive advantage in both T1 and T2 treatments, with a relative competitive ability between 0.059-0.234. When maize silage and oil sunflower are planted simultaneously, oil sunflower is the dominant crop. The net benefit of T1 treatment is the highest, with an average total net income of 85,000 Chinese Yuan/667m^2 over two years. It can be seen that diversified planting patterns can improve the yield and economic benefits per unit land area, and are a highly promising planting pattern.

Keywords: intercropping-multiple cropping; diversified planting; land equivalent ratio; economic

performance; arid area

The shortage of water resources and uneven spatial and temporal distribution are currently the main limiting factors for the development of agricultural production in China (Chai et al., 2019). Improving the water resource utilization efficiency of crops is not only an important part of the development of water-saving agriculture, but also the primary task of high-quality development of irrigation area agriculture (Cao et al., 2021). The diversified planting mode of intercropping and multiple cropping, as a long-standing and efficient intensive planting method, improves the utilization efficiency of water, nutrients, light energy and other resources through the reasonable combination of different crops in time and space. The increase in output per unit of land area and per unit of water resource has played an important role in improving the efficiency of agricultural water resource utilization and ensuring national food security (Burgess et al., 2022). The Ningxia Yellow River Irrigation Area is located in the arid and water deficient northwest plateau, with abundant solar, thermal, and wind energy resources. The region has low rainfall and high evaporation, and agricultural water mainly relies on the Yellow River for water diversion (Wang et al., 2023). In recent years, with the reduction of water inflow from the Yellow River and the limitation of water use indicators, coupled with the development of animal husbandry, there has been a serious shortage of land and water resources in the region (Qian et al., 2020). In order to achieve efficient and sustainable utilization of water resources in the Yellow River irrigation area of Ningxia and ensure the safety of food and forage crop supply, intercropping has once again attracted widespread attention from local scholars (Lu et al., 2022).

Intercropping, due to the full utilization of water, fertilizer, gas, and heat resources in farmland, can significantly increase crop yield compared to monoculture (LI et al., 2021). However, under the intercropping model of two crops, sowing width, planting density, and variety combination have a significant impact on the growth of the intercropping system (Raza et al., 2022). Currently, many scholars have explored suitable planting ratios and bands for intercropping systems such as maize-common bean (Alemayehu et al., 2017), maize-black gram/green gram/vegetable cowpea/sea/ground nut (Parimaladevi et al., 2019), maize-canopy (Khanum et al., 2019), as well as optimized fertilization under intercropping modes (Ndungu Magiroi et al., 2017), intercropping advantages (Khanal et al., 2021) and resource utilization of intercropping between two crops (Yin

et al., 2020). Various suitable intercropping planting modes and water and fertilizer management schemes have been proposed (Martin-Guay et al., 2018). There are also a lot of studies on multiple cropping, which has increased the sown area, improved grain yield, and effectively enhanced grain production capacity (Waha et al., 2020). Multiple intercropping modes have been proposed based on temperature and crop characteristics, such as intercropping vegetables after wheat (Hufnagel et al., 2020) and intercropping grass after wheat (Zimbric et al., 2020). There have also been many achievements in water use in intercropping cultivation modes of multiple crops. Research has found that under intercropping modes, there are two aspects of competition and complementarity in water use between two crops, and this relationship is mainly influenced by various factors such as crop types, irrigation and fertilization systems, planting density, spatial layout, tillage and coverage measures, and environmental factors (Yin et al., 2020). The crop variety has a significant impact on the water use efficiency of intercropping systems (Sun et al., 2021). In the soybean wheat/maize system, the soil moisture in soybean strips can be used by intercropping wheat or corn, increasing the soil water storage capacity of wheat and corn strips and improving the soil water use efficiency (Zhang et al., 2022). In the wheat maize intercropping system, wheat has stronger water competitiveness. Although the total water consumption of the intercropping system has decreased compared to monoculture, this is mainly due to the reduction in water consumption of wheat (Ma et al., 2024).

However, it can be seen that current research on intercropping and multiple cropping is still conducted separately, and the research objects are relatively single. There are few reports on the planting modes of various crops such as grain crops, forage crops, and vegetables, and there is a lack of research on the advantages of intercropping and water use status after directly intercropping crops in the same year after harvest. Therefore, this article conducts research on the intercropping advantages, water production efficiency, competitive advantages, and economic benefits of different intercropping modes for grain crops such as wheat and corn, as well as diversified planting modes such as cabbage, sorghum-sudangrass hybrid, and maize silage. Suitable intercropping planting modes and crop species are proposed for the experimental area, providing a scientific basis for the promotion of diversified planting modes in the experimental area.

1. Materials and Methods

1.1 Overview of the experimental area

The experimental base is located in Wanghong Town, Yongning County, China, with a geographical location of 37°55′ N latitude, 106°6′ E longitude, and an altitude of 1106 meters. It has a typical temperate continental climate with abundant solar and thermal resources. The average annual temperature is 9.3 ℃, the annual sunshine hours are 2974.4 hours, the average frost free period is 171 d, the average annual precipitation is 260.7 mm, and the average annual evaporation is 2013.7 mm. The soil in the experimental area is sticky loam soil, with an average field water holding capacity of 33.54% (volume percentage) and an average soil bulk density of 1.37 g/cm^3. Alkaline hydrolyzed nitrogen is 82 mg/kg, available phosphorus is 26.2 mg/kg, available potassium is 147 mg/kg, and organic matter content is 9.2 g/kg.

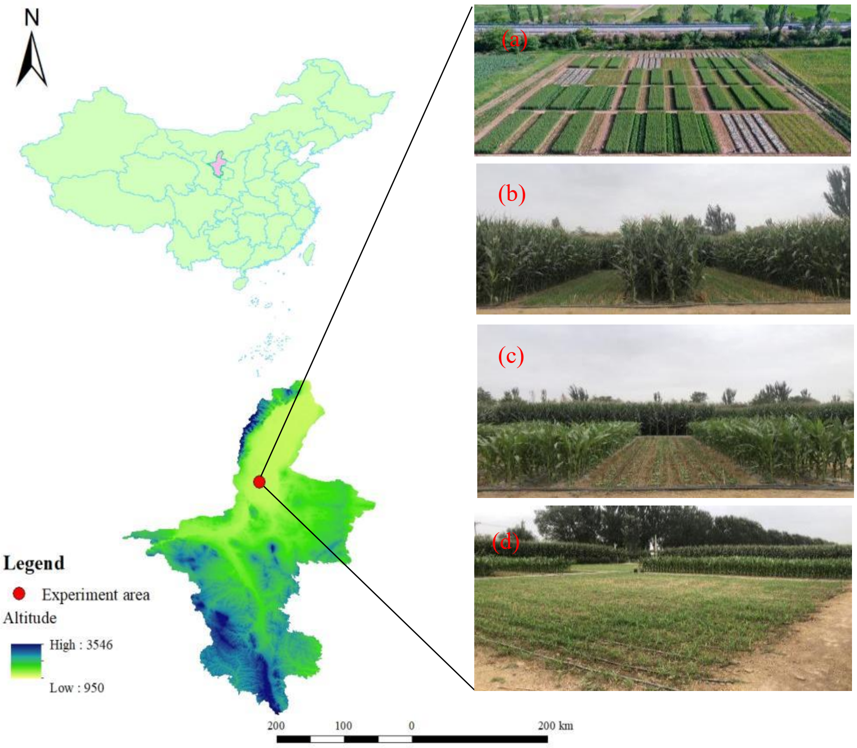

Figure 1 Location of the experimental area and test photos. A is the overall view of the experimental base, b is intercropping wheat with maize silage and replanting sorghum-sudangrass hybrid after wheat harvest, c is intercropping wheat with cabbage, replanting oil sunflower after wheat harvest, and replanting maize silage after cabbage harvest, d is single wheat, after wheat harvest, half of the land is replanted with maize silage, and half of the land is replanted with oil sunflower

1.2 Experimental design

This experiment focuses on diversified planting modes, specifically intercropping and multiple cropping. This study primarily explores the intercropping effects and water use efficiency of various crops under different planting modes. A total of 4 diversified planting modes were designed, including intercropping wheat with maize silage and replanting sorghum-sudangrass hybrid after wheat harvest (T1), the sowing area for wheat and sorghum-sudangrass hybrid is 367 m^2, and for maize silage it is 300 m^2; intercropping wheat with cabbage, replanting oil sunflower after wheat harvest, and replanting maize silage after cabbage harvest (T2), the sowing area for wheat and oil sunflower is 367 m^2, cabbage and maize silage it is 300 m^2; single wheat, after wheat harvest, half of the land is replanted with maize silage, and half of the land is replanted with oil sunflower (T3), the sowing area for wheat is 667 m^2, while for maize silage and oil sunflower it is 338.5 m^2; single maize silage (T4), the sowing area is 667 m^2. Each treatment set has 3 repetitions, and each plot has an area of 667 m^2. The irrigation method is drip irrigation, with a drip head spacing of 0.3m and a drip head flow rate of 2 L/h. The distance between drip irrigation belts for wheat is 50 cm, and other crops are arranged in a row along the belt. The sowing date, harvesting date, and irrigation conditions for each crop under the intercropping-multiple cropping are shown in Table 1.

Table 1 The sowing date, harvesting date, and irrigation conditions for each crop under the diversified planting mode of intercropping and multiple cropping

Crop	Planting type	Sowing date	Harvest date	Irrigation	
				Amount/(mm)	Frequency/(time)
Wheat	Normal planting	March 2nd	July 10th	285	6
Maize silage	Normal planting	April 15th	September 17th	277.5	8
	Multiple cropping of wheat	July 13th	September 17th	120	3
	Multiple cropping of cabbage	June 5nd	September 17th	207	6
Gaodan grass	Multiple cropping	July 13th	September 17th	120	3
Cabbage	Normal planting	March 24th	June 2nd	260	7
Oil sunflower	Multiple cropping	July 14th	September 17th	120	3

1.3 Indicators and methods

1.3.1 Observation indicators and methods

(1)Biomass: Harvest 1 m^2 of fresh ground samples of each crop during the harvest period and

bring them back to the laboratory. Use an oven to first adjust to 105 °C for wilting and then adjust to 80 °C for drying until a constant weight is achieved.

(2)Soil moisture content: Determine the soil moisture content before and after each irrigation and after rainfall using the drying and weighing method. The sampling location is at the edge row and 20 cm inward for wheat, and at the edge row and one inward row for other crops.

1.3.2Evaluating indicator

(1) Intercropping advantage

The intercropping advantage of intercropping populations is usually measured by two indicators: land equivalent ratio (LER), which represents the difference between the yield of intercropping populations and the yield of monoculture with the same crop and area. The calculation formula for LER is shown in 1-1:

$$P_{LER} = P_{LER_1} + P_{LER_2} + \ldots = I_1/Y_1 + I_2/Y_2 + \ldots \quad \text{1-1}$$

Where: P_{LER} is land equivalence ratio of intercropping groups, P_{LER_1} is the relative yield of first crop in intercropping, P_{LER_2} is relative yield of second crop in intercropping, relative yield is also known as the partial land equivalent ratio of a certain crop in an intercropping population. I_1 and Y_1 are the yield of a crop in an intercropping group and its corresponding monoculture yield, I_2 and Y_2 is the yield of another crop in the intercropping group and its corresponding monoculture yield, respectively. The formula for calculating the difference in yield between intercropping populations and monoculture with the same area of corresponding crops is:

$$Y = Y_S - (Y_1\alpha + Y_2\beta + \ldots) \quad \text{1-2}$$

Where: Y is intercropping population biomass yield, t/ha; Y_S is the sum of the biomass yields of two crops in an intercropping group, t/ha; and α and β are the proportion of each crop area in the entire test area.

(2)Inter species relative competitive ability

Evaluating the competitive ability of one crop in intercropping populations relative to the other for growth resources such as water, fertilizer, air, and heat, we use inter species relative competitiveness, calculate using equations 1-3:

$$A_{1,2} = I_1/(Y_1\alpha) - I_2/(Y_2\alpha)$$

$A_{1,2}>0$, indicating that the competitiveness of the first crop in the intercropping population is greater than that of the second crop; $A_{1,2}<0$, indicating that the competitiveness of the second crop in the intercropping population is greater than that of the first crop.

2. Results and discussion

2.1 Itercropping advantage

Advantages of intercropping under different intercropping and multiple cropping modes are shown in Table 2. Analysis shows that under intercropping and multiple cropping modes, all T1-T3 treatments increased the land equivalent ratio, with significant differences between the treatments. Especially, the T2 treatment achieved P_{LER} of 2.24 and 2.30 in 2022 and 2023, respectively. This is because the T2 treatment planted four crops, including maize silage, a crop with high water production efficiency, which greatly increased the yield of ground biomass compared to single cropping. Next is the T3 treatment, with a P_{LER} of 2.04 and 2.05 in 2022 and 2023, respectively. This model involves intercropping oil sunflower and maize silage after wheat harvest, which greatly increases biological yield compared to monoculture. Among the three intercropping modes, the T1 treatment had the smallest PLEW of 1.76 and 1.78 in 2022 and 2023, respectively, but it also greatly increased the biomass yield per unit area compared to monoculture. The total biomass yield of the T1 treatment increased by 0.66 t/667m^2 and 0.62 t/667m^2 compared to the same area of maize monoculture, an increase of 25.29% and 23.48% in 2022 and 2023, respectively. The increase in total biomass was most significant in the T2 treatment, with an increase of 2.65 t/667m^2 and 2.73 t/667m^2 compared to maize monoculture, an increase of 101.53% and 104.48%, respectively, in the two years. The T3 treatment also demonstrated a significant advantage in increasing total biomass, with an increase of 1.16 t/667m^2 and 1.2 t/667m^2 compared to monoculture maize, an increase of 44.44% and 45.45%. From the perspective of intercropping population biomass yield, the T1 and T2 treatments have significant advantages in biomass increase compared to monocropping maize on the same land area, with Y values of 0.49 t/667m^2 and 0.55 t/667m^2 in 2022, and Y values of 0.47 t/667m^2 and 0.65 t/667m^2 in 2023. However, the T3 treatment showed no significant pattern, decreasing by 0.02 t/667m^2 in 2022 and increasing by 0.03 t/667m^2 in 2023, indicating a certain degree of variability. It can be seen that through intercropping and multiple cropping, the total biomass yield per unit area can be significantly

increased, and the biodiversity of farmland is also increased, with obvious intercropping advantages and yield-increasing effects.

Table 2　Advantages of intercropping under different intercropping and multiple cropping modes in 2022-2023

Years	Modes	Crops	Cultivated area/(m^2)	Biomass/ (t/Cultivated area)	P_{LEWi}	P_{LEW}	Y/ (t/Cultivated area)
2022	T1	Wheat	367	0.64±0.12	0.57±0.05	1.76 ± 0.25^{Cc}	0.49 ± 0.05^{Bb}
		Maize silage	300	1.55±0.21	0.59±0.06		
		sorghum-sudan grass hybrid	367	1.08±0.19	0.60±0.12		
	T2	Wheat	367	0.71±0.09	0.63±0.08	2.24 ± 0.18^{Aa}	0.55 ± 0.04^{Aa}
		Cabbage	300	1.74±0.32	0.54±0.11		
		Maize silage	300	1.23±0.27	0.47±0.07		
		Oil sunflower	367	1.58±0.14	0.60±0.12		
	T3	Wheat	667	1.18±0.08	1.06±0.29	2.04 ± 0.12^{Bb}	-0.02 ± 0.05^{Cc}
		Maize silage	338.5	1.40±0.23	0.53±0.13		
		Oil sunflower	338.5	1.19±0.16	0.45±0.15		
	T4	Maize silage	667	2.61±0.12			
2023	T1	Wheat	367	0.66±0.08	0.59±0.05	1.78 ± 0.18^{Cc}	0.47 ± 0.07^{Bb}
		Maize silage	300	1.48±0.11	0.56±0.04		
		sorghum-sudan grass hybrid	367	1.12±0.14	0.63±0.11		
	T2	Wheat	367	0.76±0.09	0.68±0.06	2.30 ± 0.22^{Aa}	0.65 ± 0.11^{Aa}
		Cabbage	300	1.78±0.21	0.55±0.05		
		Maize silage	300	1.22±0.12	0.46±0.08		
		Oil sunflower	367	1.61±0.08	0.61±0.14		
	T3	Wheat	667	1.16±0.07	1.03±0.15	2.05 ± 0.19^{Bb}	0.03 ± 0.01^{Cc}
		Maize silage	338.5	1.43±0.11	0.54±0.09		
		Oil sunflower	338.5	1.25±0.09	0.47±0.05		
	T4	Maize silage	667	2.64±0.23			

Note: The results of comparing the land equivalent ratio in the table with that of a single maize variety, where uppercase letters indicate 5% significance and lowercase letters indicate 1% significance.

2.2 Water production efficiency

To evaluate the biological yield and economic benefits of water input per unit, the biological yield per cubic meter of water and the output value per cubic meter of water were used to analyze and evaluate the water production benefits under the intercropping-multiple cropping diversified planting model (Figure 2). The analysis shows that there are significant differences in biomass and output value generated per unit of water input under different planting modes. The T3 treatment had the highest biomass yield per cubic meter of water, reaching 12.6 kg/m^3 in 2022 and 12.7 kg/m^3 in 2023. It was followed by T1 and T2, while the T4 treatment had the lowest yield. This indicates that intercropping and multiple cropping planting modes, combined with efficient water-saving irrigation, significantly improve the production capacity of biomass yield per cubic

meter of water. Another indicator for evaluating the superiority of water use efficiency in intercropping and multiple cropping models is the output value per cubic meter of water, which is also an important factor affecting the promotion of diversified planting models. The output value of T1 treatment for one cubic meter of water was the highest, reaching 16.73 Chinese Yuan/m^3 in 2022 and 16.88 Chinese Yuan/m^3 in 2023. It was followed by T2 and T3, while the T4 treatment had the lowest output value, mainly because the control T4 was flood irrigation with a larger amount of water, and only one crop of maize silage was planted, resulting in a decrease in the output value of one cubic meter of water. On the other hand, T1 had a higher output value due to both grain and grass production.

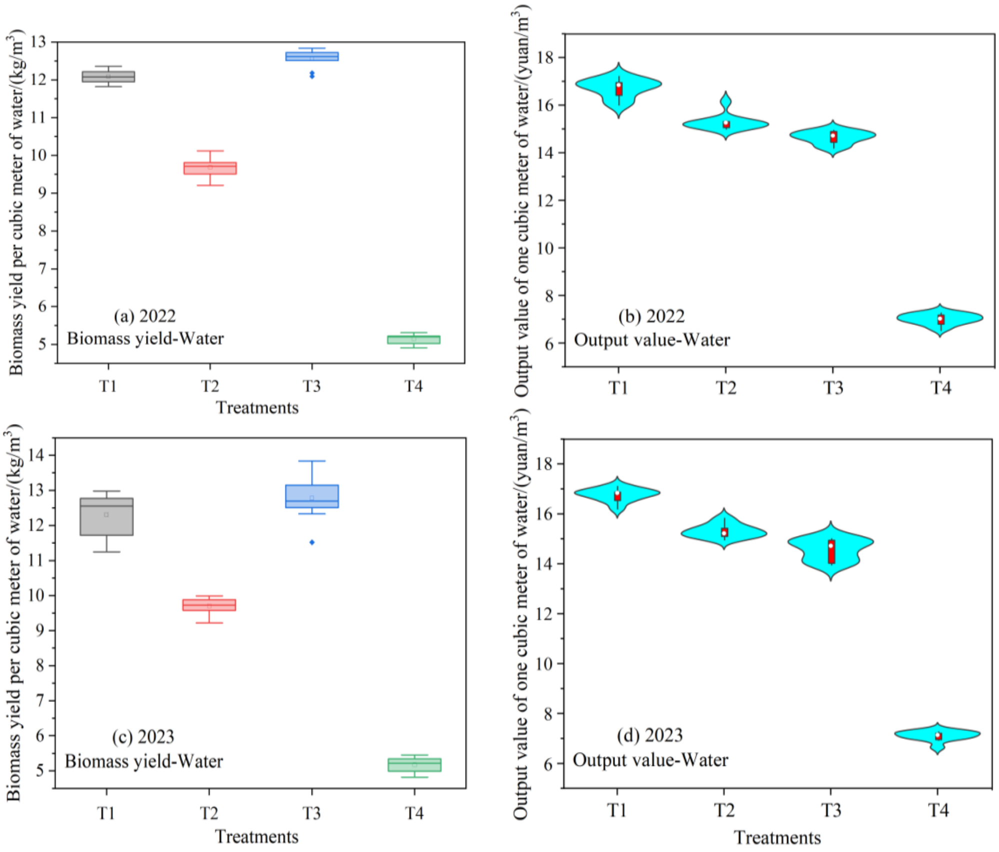

Figure 2 The biological yield (a) and output value (b) per cubic meter of water in2022-2023

2.3 Inter species relative competitive ability

When two crops are intercropped, different crops compete for water, fertilizer, and light and heat resources. Due to differences in sowing time, root distribution, and aboveground growth, the two crops may exhibit competitive advantages or disadvantages, which can sometimes be reversed. The interspecies relative competitive ability of two intercropping crops under different planting

modes is shown in Figure 3. From the final biological yield, maize has a competitive advantage compared to wheat and the sorghum-sudangrass hybrid that is replanted after wheat. The interspecies relative competitive ability is 0.292 and 0.222 in 2022, 0.177 and 0.107 in 2023, respectively. This is because under this cultivation mode, maize is sown earlier, with a wide range of root distribution and higher plant height, which can better obtain growth resources above and below ground. After intercropping cabbage and wheat, there was no significant trend. The cabbage showed a competitive advantage in 2022 (the interspecies relative competitive ability was 0.039), while wheat showed a competitive advantage in 2023 (the interspecies relative competitive ability was 0.014). When intercropping maize and sunflowers, maize showed a resource competitive advantage compared to sunflowers under T2 cultivation modes, with relative interspecies competitive abilities of 0.041 in 2022, and 0.078 in 2023, respectively. Due to the shorter growth period of cabbage and earlier sowing of maize in multiple cropping, the resource competitiveness of maize is greater than that of sunflower. However, when maize silage and oil sunflower were sown simultaneously (T3), sunflower showed a competitive advantage in resources, with interspecies relative competitive ability of 0.164 in 2022 and 0.137 in 2023. In summary, when maize silage is intercropped with other crops, it generally exhibits a competitive advantage in resources. Cabbage and wheat have similar competitive abilities for resources, and it is necessary to reasonably match the crop varieties of the intercropping population according to the water and fertilizer resources.

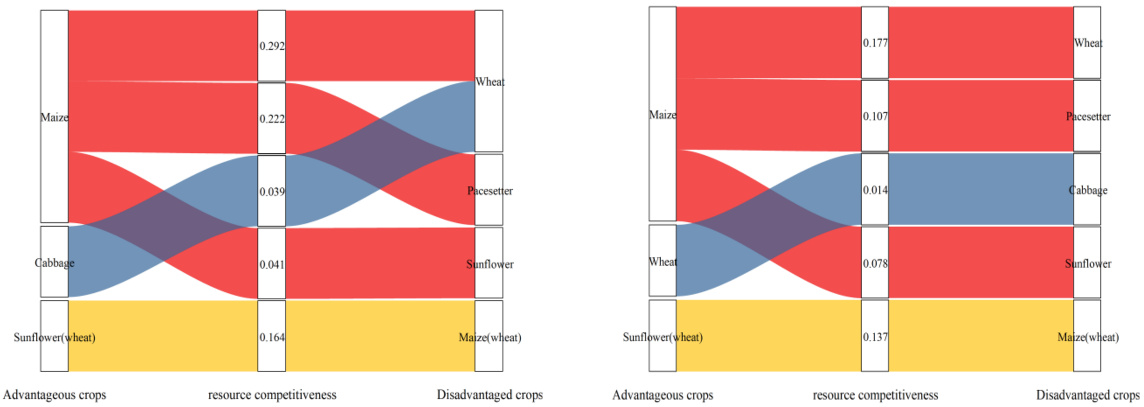

Figure 3 The inter species relative competitive ability of two intercropping crops in 2022(a) and 2023(b), Sunflowers (wheat) and corn (wheat) were replanted after T3 treatment with wheat

2.4 Output value of each mode

Due to differences in market demand, the economic efficiency of different planting modes will also show significant differences (Table 3). Analysis shows that T1 treatment has the highest net benefit, with a total net income of 84,800 Chinese Yuan/ha for all crops in 2022, and 85,100 Chinese Yuan/ha in 2023, followed by T2 and T3. T1-T3 treatments have shown an increase of 78.51%-178.51% in 2022 and 78.26%-146.67% in 2023, respectively, in comparison to T4 (control). Among all crops, maize silage has the highest net benefit, with the net income reaching 2,430-57,600 Chinese Yuan/ha within two years in each planting mode, and the highest net benefit is in T1 treatment, because maize silage has significant competitive advantages over wheat and sorghum-sudangrass hybrid under T1 treatment. At the same time, maize silage will harvest both grains and straw in this mode, thus having high economic benefits. Among wheat cultivated under various modes, T3 treatment has the lowest economic benefits with a net income of only 17,900 Chinese Yuan/ha and 17,100 Chinese Yuan/ha in 2022-2023, followed by T1 treatment (19,700 Chinese Yuan/ha and 19900 Chinese Yuan/ha), and T2 treatment has the highest net income for wheat (22600 Chinese Yuan/ha and 23100 Chinese Yuan/ha). This may be related to the variety of wheat used in intercropping. Compared with maize silage, wheat has a stronger interspecific competition ability when grown with cabbage. Among different crops, the economic benefits of sorghum-sudangrass hybrid and cabbage are the lowest. Specifically, sorghum-sudangrass hybrid had a net income of 7,500 Chinese Yuan/ha in 2022 and 7,100 Chinese Yuan/ha in 2023, while cabbage had a net income of 12,700 Chinese Yuan/ha in 2022 and 12,100 Chinese Yuan/ha in 2023. This is mainly due to the lower yield of sorghum-sudangrass hybrid and the higher input for cabbage, resulting in lower net incomes for these two crops. In summary, in intercropping and multiple cropping modes, due to the water replenishment relationship between different crops, they can improve economic benefits to a certain extent compared to monoculture, making these modes potential cultivation options.

Table 3 Economic benefits of different planting modes in 2022-2023

Years	Modes	Crops	Output value of various crops/(Ten thousand yuan/ha)	Input value of various crops/(Ten thousand yuan/ha)	Net income for each mode/(Ten thousand Chinese Yuan/ha)
2022	T1	Wheat	3.20±0.12	1.23±0.08	8.48 ± 0.22^{Bb}
		Maize silage	7.32±0.32	1.56±0.11	
		Gaodan grass	1.38±0.09	0.63±0.05	
	T2	Wheat	3.51±0.31	1.25±0.09	8.39 ± 0.26^{Aa}
		Cabbage	3.60±0.28	2.33±0.13	
		Maize silage	4.23±0.38	0.90±0.18	

		Oil sunflower	2.16 ± 0.16	0.63 ± 0.12	
		Wheat	3.03 ± 0.11	1.24 ± 0.09	
	T3	Maize silage	3.07 ± 0.23	0.64 ± 0.10	6.23 ± 0.18^{Cc}
		Oil sunflower	2.65 ± 0.17	0.64 ± 0.08	
	T4	Maize silage	5.01 ± 0.22	1.52 ± 0.22	3.49 ± 0.11^{Dd}
2023	T1	Wheat	3.24 ± 0.16	1.25 ± 0.07	8.51 ± 0.28^{Bb}
		Maize silage	7.41 ± 0.24	1.62 ± 0.09	
		Gaodan grass	1.35 ± 0.15	0.64 ± 0.12	
	T2	Wheat	3.54 ± 0.22	1.23 ± 0.08	8.43 ± 0.31^{Aa}
		Cabbage	3.58 ± 0.18	2.37 ± 0.17	
		Maize silage	2.33 ± 0.11	0.95 ± 0.08	
		Oil sunflower	2.21 ± 0.16	0.68 ± 0.13	
	T3	Wheat	2.98 ± 0.21	1.27 ± 0.11	6.15 ± 0.22^{Cc}
		Maize silage	3.14 ± 0.24	0.68 ± 0.07	
		Oil sunflower	2.64 ± 0.18	0.66 ± 0.15	
	T4	Maize silage	4.98 ± 0.19	1.53 ± 0.19	3.45 ± 0.14^{Dd}

Note: The results of comparing the land equivalent ratio in the table with that of a single maize variety, where uppercase letters indicate 5% significance and lowercase letters indicate 1% significance.

3. Disscution

In intercropping planting mode, due to the complementary effects of edge row effect and water, fertilizer, light and heat resources, the crop yield for intercropping is generally higher than that of monoculture with the same land area (Wang et al., 2022). At present, research on the advantages of intercropping mainly focuses on the maize soybean intercropping model (Feng et al., 2020; Raza et al., 2021), only a small amount of research has been reported on other intercropping modes. Under rain-fed conditions in semi-humid areas, strip intercropping of wheat/maize can significantly improve land use efficiency and total land yield. In this mode, the increase in yield of the entire planting pattern is mainly due to the increase in wheat yield, which can increase by more than 35%, while corn yield remains consistent with the monoculture mode (Li et al., 2020). The results of this study indicate that when wheat and maize silage are intercropped, both show increased yields compared to monoculture (Table 2). This is because the distance between the wheat and maize silage rows was expanded to 30cm during sowing, which first minimized the stress effect of wheat on the early growth of maize silage. In the intercropping mode of corn/beans or wheat/beans, the LER is generally less than 1.5 (Xu et al., 2020; Abusief et al., 2021). However, in this study, the LER of each mode ranged from 1.76-2.3 in two years. This is mainly because the planting mode in this study involves various forms such as intercropping and multiple cropping, changing one crop to two crops, thus greatly improving the LER of each mode. Under the intercropping mode between two crops, due to the mutual influence between the aboveground and underground parts, there is generally a competitive relationship between the two crops in terms of

growth resources such as water, fertilizer, gas, and heat (Zhang et al., 2021). In the traditional corn/wheat intercropping system, due to the early sowing of wheat, it often has an advantage in intercropping during growth. Although corn may recover to a certain extent in the later stage, it still remains at a disadvantage in intercropping (Wang et al., 2022). However, in this study, we appropriately increased the distance between the wheat edge row and the corn edge row to reduce the resource competition of wheat for early growth of corn, and through appropriate water and fertilizer management in the later stage, the growth of corn was restored. After calculation, it was found that corn had a competitive advantage over wheat at maturity (Figure 3). There is relatively little research on the intercropping of maize silage and sorghum-sudangrass hybrid. When these two crops are intercropped, corn has a significant intercropping advantage because sorghum-sudangrass hybrid is replanted after wheat harvest, and corn is in the tasseling silking stage, with a larger plant height and developed root system, which can absorb more water and fertilizer resources. In the intercropping system between maize and sunflowers, sunflowers are generally the competitive advantage crop (Nassab et al., 2011). In this study, sunflowers were found to have a competitive advantage in the intercropping system of corn and sunflowers after wheat harvest, which is consistent with previous research results. However, in T3 treatment, corn has a competitive advantage, which may be due to the earlier harvest of cabbage than wheat in the previous crop, the earlier time of corn replanting, and the significant growth advantage of corn when sunflowers emerge (Figure 3). The intercropping system can achieve diversified planting according to the market, which increases the ability to resist market risks compared to single planting. Due to the different types of crops planted, the economic benefits also vary greatly, ranging from 10,000 to 95,000 yuan/ha (Yang et al., 2018; Ouyang et al., 2017). The average net benefit of T1-T3 treatment in this study ranges from 61,900 to 85,000 yuan/ha over two years (Table 3). It can be seen that under the intercropping multiple cropping planting mode, not only can the output efficiency of land be improved, but also the economic benefits of agricultural production can be enhanced. However, it is necessary to choose a reasonable intercropping planting mode based on local planting habits, market demand, and production conditions.

Funding: This work was supported by the National Key Research & Development Program of China (2021YFD1900600, 2022YFG1900205); the Key Research & Development Program of

Ningxia Hui Autonomous Region (2021BEF02034); the Ningxia Natural Science Foundation Project (2022AAC03725)

Conflicts of Interest: The authors declare no conflict of interest.

4. Conclusion

This study compared the biological yield and economic benefits of intercropping and multiple cropping diversified planting modes, and analyzed the optimal planting mode of intercropping and multiple cropping in the Yellow River irrigation area of Ningxia. The results showed that due to the implementation of multiple crop planting on the same land area within a year, as well as the mixed planting of various crops such as vegetables, forage, and grains, not only did the grain yield greatly increase, but also the harvest of vegetables and forage was achieved, supporting regional food security and the rapid development of animal husbandry.

Reference

Chai, J., Wang, Z., Yang, J., & Zhang, L. (2019). Analysis for spatial-temporal changes of grain production and farmland resource: Evidence from Hubei Province, central China. Journal of cleaner production, 207, 474-482.

Cao, X., Xiao, J., Wu, M., Zeng, W., & Huang, X. (2021). Agricultural water use efficiency and driving force assessment to improve regional productivity and effectiveness. Water Resources Management, 35(8), 2519-2535.

Burgess, A. J., Cano, M. E. C., & Parkes, B. (2022). The deployment of intercropping and agroforestry as adaptation to climate change. Crop and Environment, 1(2), 145-160.

Wang, W., Jing, H., Guo, X., Dou, B., & Zhang, W. (2023). Analysis of Water and Salt Spatio-Temporal Distribution along Irrigation Canals in Ningxia Yellow River Irrigation Area, China. Sustainability, 15(16), 12114.

Qian, Z. H., Han, B. F., Liu, Z. T., Cai, W., Fu, B. Z., & Ma, H. B. (2020). Effects of infiltration irrigation on growth characters and water use efficiency of alfalfa in the Ningxia Yellow River diversion irrigation area. Acta Prataculturae Sinica, 29(3), 147.

Lu, X. (2022). Effect of intercropping soybean on the diversity of the rhizosphere soil arbuscular mycorrhizal fungi communities in wheat fields. CLEAN – Soil, Air, Water, 50(6), 2100014.

Li, X. F., Wang, Z. G., Bao, X. G., Sun, J. H., Yang, S. C., Wang, P., ... & Li, L. (2021). Long-term increased grain yield and soil fertility from intercrop. Nature Sustainability, 4(11), 943-950.

Raza, M. A., Yasin, H. S., Gul, H., Qin, R., Mohi Ud Din, A., Khalid, M. H. B., ... & Yang, W. (2022). Maize/soybean strip intercrop** produces higher crop yields and saves water under semi-arid conditions. Frontiers in Plant Science, 13, 1006720.

Alemayehu, A., Tamado, T., Nigussie, D., Yigzaw, D., Kinde, T., & Wortmann, C. S. (2017). Maize–common bean intercropping to optimize maize-based crop production. The Journal of Agricultural Science, 155(7), 1124-1136.

Parimaladevi, C., Ramanathan, S. P., & Suresh, S. (2019). Evaluation of maize based intercropping systems in

Thamirabarani basin of Tamil Nadu. Journal of Pharmacognosy and Phytochemistry, 8(3), 4051-4056.

Khanum, M. M., Bazzaz, M. M., Ahmed, B., Huda, M. S., & Hossain, M. A. (2019). Intercropping of cabbage with maize. Bangladesh Agronomy Journal, 22(1), 115-120.

Ndungu-Magiroi, K. W., Wortmann, C. S., Kibunja, C., Senkoro, C., Mwangi, T. J., Wamae, D., ... & Msakyi, J. (2017). Maize-bean intercrop response to nutrient application relative to maize sole crop response. Nutrient Cycling in Agroecosystems, 109, 17-27.

Khanal, U., Stott, K. J., Armstrong, R., Nuttall, J. G., Henry, F., Christy, B. P., ... & O'Leary, G. J. (2021). Intercropping—Evaluating the Advantages to Broadacre Systems. Agriculture, 11(5), 453. doi.org/10.3390/agriculture11050453

Yin, W., Chai, Q., Zhao, C., Yu, A., Fan, Z., Hu, F., ... & Coulter, J. A. (2020). Water utilization in intercropping: A review. Agricultural Water Management, 241, 106335.

Martin-Guay, M. O., Paquette, A., Dupras, J., & Rivest, D. (2018). The new green revolution: sustainable intensification of agriculture by intercrop**. Science of the total environment, 615, 767-772.

Waha, K., Dietrich, J. P., Portmann, F. T., Siebert, S., Thornton, P. K., Bondeau, A., & Herrero, M. (2020). Multiple cropping systems of the world and the potential for increasing cropping intensity. Global Environmental Change, 64, 102131.

Hufnagel, J., Reckling, M., & Ewert, F. (2020). Diverse approaches to crop diversification in agricultural research. A review. Agronomy for Sustainable Development, 40(2), 14.

Zimbric, J. W., Stoltenberg, D. E., & Picasso, V. D. (2020). Effective weed suppression in dual-use intermediate wheatgrass systems. Agronomy Journal, 112(3), 2164-2175.

Yin, W., Chai, Q., Zhao, C., Yu, A., Fan, Z., Hu, F., ... & Coulter, J. A. (2020). Water utilization in intercropping: A review. Agricultural Water Management, 241, 106335.

Sun, T., Zhao, C., Feng, X., Yin, W., Gou, Z., Lal, R., ... & Zhang, W. (2021). Maize-based intercropping systems achieve higher productivity and profitability with lesser environmental footprint in a water-scarce region of northwest China. Food and Energy Security, 10(1), e260.

Zhang, H., Shi, W., Ali, S., Chang, S., Jia, Q., & Hou, F. (2022). Legume/maize intercropping and N application for improved yield, quality, water and n utilization for forage production. Agronomy, 12(8), 1777.

Ma, L., Li, Y., Wu, P., Zhao, X., Chen, X., & Gao, X. (2024). Evaluating water use advantage of wheat/maize relay intercropping under rainfed condition based on same period. Plant and Soil, 499(1), 9-21.

Wang, W., Li, M. Y., Gong, D. S., Zhou, R., Khan, A., Zhu, Y., ... & Xiong, Y. C. (2022). Water use of intercropped species: Maize-soybean, soybean-wheat and wheat-maize. Agricultural Water Management, 269, 107690.

Feng, L., Raza, M. A., Shi, J., Ansar, M., Titriku, J. K., Meraj, T. A., ... & Yang, W. (2020). Delayed maize leaf senescence increases the land equivalent ratio of maize soybean relay intercropping system. European Journal of Agronomy, 118, 126092.

Raza, M. A., Gul, H., Wang, J., Yasin, H. S., Qin, R., Khalid, M. H. B., ... & Yang, W. (2021). Land productivity and water use efficiency of maize-soybean strip intercropping systems in semi-arid areas: A case study in Punjab Province, Pakistan. Journal of Cleaner Production, 308, 127282.

Li, Y., Ma, L., Wu, P., Zhao, X., Chen, X., & Gao, X. (2020). Yield, yield attributes and photosynthetic physiological characteristics of dryland wheat (*Triticum aestivum* L.)/maize (Zea mays L.) strip intercropping. Field Crops Research, 248, 107656.

Xu, Z., Li, C., Zhang, C., Yu, Y., van der Werf, W., & Zhang, F. (2020). Intercropping maize and soybean increases efficiency of land and fertilizer nitrogen use; A meta-analysis. Field Crops Research, 246, 107661.

Abusief, H. M., Alhaddad, A., Mukassabi, T. A., Alawami, M. O., & Al-Arifi, H. O. A. A. (2021). Land equivalent coefficient of intercropped varieties of wheat (Triticum aestivum) with pea (Pisum sativum) in two sites of

Qandulah and Al-Baidain, Libya. Research on Crops, 22(3), 483-491.

Zhang, W. P., Gao, S. N., Li, Z. X., Xu, H. S., Yang, H., Yang, X., ... & Li, L. (2021). Shifts from complementarity to selection effects maintain high productivity in maize/legume intercropping systems. Journal of Applied Ecology, 58(11), 2603-2613.

Wang, W., Li, M. Y., Gong, D. S., Zhou, R., Khan, A., Zhu, Y., ... & Xiong, Y. C. (2022). Water use of intercropped species: Maize-soybean, soybean-wheat and wheat-maize. Agricultural Water Management, 269, 107690.

Nassab, A. D. M., Amon, T., & Kaul, H. P. (2011). Competition and yield in intercrops of maize and sunflower for biogas. Industrial Crops and Products, 34(1), 1203-1211. doi.org/10.1016/j.indcrop.2011.04.015

Yang, C., Fan, Z., & Chai, Q. (2018). Agronomic and economic benefits of pea/maize intercrop** systems in relation to N fertilizer and maize density. Agronomy, 8(4), 52.

Ouyang, C., Wu, K., An, T., He, J., Zi, S., Yang, Y., & Wu, B. (2017). Productivity, economic, and environmental benefits in intercropping of maize with chili and grass. Agronomy Journal, 109(5), 2407-2414.
