# Supplementary material for: Water production efficiency and economic benefits under diversified planting modes of intercropping-multiple cropping in arid regions
Source: PLoS One. 2025 Mar 10;20(3):e0318540. doi: 10.1371/journal.pone.0318540 (PMC11892835; doi:10.1371/journal.pone.0318540)
Supplement: Attachment — Submitted filename: Response to Reviewers.docx [file pone.0318540.s005.docx]

Dear editor:

Thank you and the two anonymous reviewers greatly for the insightful comments and suggestions on the early version of the manuscript. We have taken all of them seriously and had revisions accordingly. Our revisions and responses are detailed below in blue. Your reconsiderations are highly appreciated!

Sincerely yours

Best Regards

**Comprehensive opinions of the editorial department**

**Question 1:** In the introduction, is not sufficient delving into background review to justify importance of the study (more literature required, preferably no older than 5 years.

**Response:** Thank you to the reviewer for the careful review. According to the reviewer's comments, the introduction section has been reorganized. Firstly, the severe competition between grain crops and forage crops in the research area for land, water, and other resources has been analyzed, highlighting the threat of resource scarcity to regional food security and human health. The second paragraph introduces the advantages of intercropping and multiple cropping as diversified planting modes for land and water resource utilization. It summarizes the current research progress, main conclusions of these planting modes, and discusses the research conducted on them in the Ningxia Yellow River Irrigation Area. The third paragraph outlines the shortcomings in current research on intercropping and multiple cropping modes, and presents the research content and significance of this study. Please refer to the revised draft for specific modifications.

**Question 2:** In Methods, although one reviewer commended the general model applied in materials and methods, the other thought that there would better be need to Include a conceptual or diagrammatic framework outlining the experimental protocol.

**Response:** Thank you to the reviewer for the careful review. This study mainly explored the water production efficiency and planting benefits of different intercropping and multiple cropping modes for grain crops, forage crops, and vegetables. Three diversified planting modes were designed, with sole maize serving as the control. The theoretical basis primarily lies in the intercropping advantage and relative competitive ability of diversified planting modes. The evaluation indicators encompass water production efficiency and output value. The main objective is to identify suitable intercropping and multiple cropping modes for the Ningxia Yellow River Irrigation Area and similar ecological regions. The diagrammatic framework and evaluation index of experimental protocol is shown in Figure 2. Please refer to the revised manuscript for specific modifications.

Figure 2 The diagrammatic framework and evaluation index of experimental protocol

**Question 3:** Also the study design needs to be more clearly out-layed and the research objectives/questions presented more clearly to address gaps in research that these questions seek to address. There is also need for a clear (set of) hypotheses, including the authors’ own expectations of what the findings might be (based on that/those hypotheses).

**Response:** Thank you to the reviewer for the careful review. The research objective of this study is to screen suitable diversified planting patterns for the Yellow River irrigation area in Ningxia and other similar ecological areas. The research objective is supplemented in lines 87-90 of the introduction. The supplementary content is as follows “The research objective of this study is to screen for intercropping-multiple cropping planting models that can simultaneously produce grain crops and forage crops in the Yellow River irrigation area of Ningxia, and have high water production efficiency and planting benefits”.

The basic hypothesis of this study “ To improve water use efficiency, it is necessary to increase the planting of corn, and in order to increase dry matter yield, Sorghum-sudangrass hybrid should be replaced by oil sunflower for replanting. The economic benefits should be analyzed based on the market prices of forage and vegetables.” This hypothesis has been supplemented in Section 1.2, this study explores the dry matter yield and economic benefits under different intercropping and multiple cropping diversified planting modes based on this hypothesis.

**Question 4:** For units, the authors need to settle for either metric (such as m) or hectares throughout.

**Response:** Thank you to the reviewer for the careful review. According to the review comments, the units of the entire text have been sorted out and uniformly modified to international metric units, especially all units related to production in section 2.1 Itercropping Advantage have been modified. Please refer to the revised draft for specific modifications.

**Question 5:** In the results, there is need to Include a clear outline of the merits of the range of experimental treatments i.e. show which treatments ranked higher than which

**Response:** Thank you to the reviewer for the careful review. From the results of two years, T2 had the highest P_LER_ among all planting modes, followed by T3 and T1. The total biological yield of T2 was the highest, followed by T3 and T1, while T4 treatment had the lowest. The most significant intercropping population biomass yield advantages were T2 and T1, while T3 was not significant compared to monoculture. The biological yield of per cubic meter of water was highest in T3, followed by T1 and T2, and lowest in T4 treatment. The biological yield of per cubic meter of water was highest in T1, followed by T2 and T3, and lowest in T4 treatment. The output value per unit area was also highest for T1, followed by T2 and T3, and lowest for T4 treatment. The comparison results of different planting modes and indicators have been added to the corresponding section of the revised manuscript, and are represented in blue font.

**Question 6:** In the discussion, the authors need to adopt a logical sequence approach in discussing the results obtained, laying the greatest emphasis on the key findings, and also putting the results in the context of existing literature (in other words comparing with other studies elsewhere).

**Response:** Thank you to the reviewer for the careful review. Based on the opinions of the reviewers, the discussion section has been reorganized and divided into sections according to the content of the revised manuscript. The emphasis is on the importance of heat in diversified planting modes, the yield of intercropping crops, land equivalent ratio, the relative competitiveness between intercropping crops and the economic benefits in intercropping and multiple cropping modes were compared with existing research. Please refer to the revised draft for details.

**Question 7:** With regards to references, the authors should closely check for compliance with PLOS ONE guidelines

**Response:** Thank you to the reviewer for the careful review. The references in the revised manuscript have been modified according to the guidelines.

**Reviewer # 1 Questions and our responses**

**Question 1:** The abstract needs to be revised extensively. Please find comments in attached pdf file.

**Response:** Thank you to the reviewer for the careful review. According to the comments of the reviewers, the abstract has been comprehensively revised. In the experimental method, we monitored the soil moisture content of the experimental area and introduced it in section 1.3 Indicators and Methods. Please refer to the revised manuscript of the paper for details.

**Question 2:** Introduction，The introduction is not providing enough information on the research background and research gap. The authors are advised to add more relevant literature to introduce the knowledge gap. Few concerns regarding this PArt:

1. write the heading ''Introduction"

2. terms of intercropping, replanting, multiple cropping should be clearly mentioned. in some references author is talking about intercropping but the reference given is of rely-intercropping.

3. Author should be clear about all the terms regarding intercropping. please revisit the text and do not mix termonologies.

**Response:** Thank you to the reviewer for the careful review. The introduction has been revised according to the reviewer's comments. The first paragraph focuses on the background and importance of the research. The second paragraph summarized the current research progress in crop combination, water use, yield, and other aspects of intercropping and multiple cropping modes. The third paragraph analyzed the shortcomings and existing problems of the research, particularly in the Yellow River irrigation area of Ningxia, and then introduced the main content and significance of this study. Additionally, terms such as "intercropping", "replanting" and other related terms mentioned in the introduction have been unified and standardized. Please refer to the revised draft for details.

**Question 3:** experimental design needs improvement in clarity. Please show cogrowth periods (figure) of all treatments to make it understandable for readers. have you planted sole maize silage and sole oil sunflower ? if no, then why?

**Response:** Thank you to the reviewer for the careful review. Table 1 has been modified to display the sowing date, harvest date, and irrigation schedule for each crop, according to different treatment formats. In addition, the T4 is monoculture and flood irrigation of maize silage, which is the main crop and irrigation method in the Yellow River irrigation area of Ningxia and is used as a control for comparison. All other crops have the monoculture mode as the basis for calculating the land equivalent ratio (LER), which was not treated separately in this experiment. Instead, the monoculture mode planted by farmers adjacent to the experimental plot was monitored and used for calculations. The modified content of Table 1 is as follows:

Table 1 The sowing date, harvesting date, and irrigation scheduling of each crop for different experimental treatments

| Treatments | Crop | Planting type | Sowing date | Harvest date | Irrigation | |
| --- | --- | --- | --- | --- | --- | --- |
|  |  |  |  |  | Amount/  (mm) | Frequency/(time) |
| T1 | Wheat | Normal planting | March 2nd | July 10th | 285 | 6 |
|  | Maize silage | Normal planting | April 15th | September 17th | 277.5 | 8 |
|  | Sorghum-sudangrass hybrid | Multiple cropping | July 13th | September 17th | 120 | 3 |
| T2 | Wheat | Normal planting | March 2nd | July 10th | 285 | 6 |
|  | Cabbage | Normal planting | March 24th | June 2nd | 260 | 7 |
|  | Maize silage | Multiple cropping of wheat | July 13th | September 17th | 120 | 3 |
|  | Oil sunflower | Multiple cropping of cabbage | June 5nd | September 17th | 207 | 6 |
| T3 | Wheat | Normal planting | March 2nd | July 10th | 285 | 6 |
|  | Maize silage | Multiple cropping | July 13th | September 17th | 120 | 3 |
|  | Oil sunflower | Multiple cropping | July 14th | September 17th | 120 | 3 |
| T4 | Maize silage | Normal planting | April 15th | September 17th | 520 | 5 |

**Question 4:**this configuration is not mentioned in the material and method. Please add all the configurations of sole, intercropping and multiple cropping configurations in the material and method with respect to plant*plant and row*row distance.

**Response:** Thank you to the reviewer for the careful review. According to the reviewer's comments, planting methods for different crops in interplanting and multiple planting modes have been added to Section 1.2, "Experimental Design," including planting rows, row spacing, and distance between two crops. Please refer to the revised manuscript for specific modifications.

**Question 5:** I strongly recommend the authors to write results with exact significant values and then calculate percentage differences between best and worst treatments. And please recheck the results as author wrote PLER in the result and discussion section while in the associated table it is written PLEW. Please review the whole paper for consistency of terminologies and unites.
**Response:** Thank you to the reviewer for the careful review. The revised manuscript has added the relative sizes between each treatment and the percentage differences between different treatments in the results section. In addition, P_LEW_ in Table 2 is a spelling error and has been corrected. Please refer to the revised draft for details.

**Question 6:** I think authors should rethink what they write in the first paragraph and only summarize the main findings in view of the research questions. After this, authors can explore different aspects of the work in subsequent paragraphs and explain how their findings expand the envelope of knowledge, but first of all, authors simply need to state the main results without discussing their why and how or the relationships to the literature. First of all, the reader needs a clear statement on what the study found. Moreover, it is suggested to discuss the main results in a logical way.
**Response:** Thank you to the reviewer for the careful review. According to the review comments, the discussion section has been revised. In the first paragraph of the discussion, the main findings and conclusions of this study are summarized, and in the following paragraphs, the findings of this study are compared with existing reports by indicators. The same and different viewpoints as this study are compared, and the reasons for the different conclusions are analyzed. Please refer to the revised manuscript of the paper for details.

**Question 7:** Authors Read following paper on soil water balance and may include in citation.
Yi J, Li H, Zhao Y, Shao M, Zhang H,... Liu M. Assessing soil water balance to optimize irrigation schedules of flood-irrigated maize fields with different cultivation histories in the arid region. Agricultural Water Management. 2022;265:107543. https://doi.org/10.1016/j.agwat.2022.107543

**Response:** Thank you to the reviewer for the careful review. The article mentioned by the reviewer elucidates the importance of soil moisture for crop growth. In intercropping and multiple cropping systems, different crops often exhibit competitive advantages or disadvantages due to their competition for water, fertilizer, and other resources. In arid regions, soil moisture is a crucial factor determining the growth status of crops. Therefore, the article was cited in the discussion section to explain the underlying reasons for the competitive relationships observed among intercropped crops.

**Reviewer # 2 Questions and our responses**

**Question 1:** Line 24: 16.81 RMB/m^3^. In fact, I would advise the author to convert to net benifit per hectare.
**Response:** Thank you to the reviewer for the careful review. The manuscript mainly discusses the total biomass, land equivalent ratio, water production efficiency, interspecies relative competitive ability, and output value under different intercropping and multiple planting modes. This section introduces the biological yield and output value of different modes of water per cubic meter, and therefore represents the benefits per cubic meter of water. The net income per hectare is highlighted in section 2.4 of the manuscript.

**Question 2:** Line 28: m^2^? In the previous paragraph it is m^3^.
**Response:** Thank you to the reviewer for the careful review. This sentence introduces that the planting mode of T1 has the highest net income, with a net income of 84800 Chinese Yuan/ha in 2022, 85100 Chinese Yuan/ha in 2023, and an average of 84950 Chinese Yuan/ha for the two years. It has been revised in line 28 of the abstract.

**Question 3:** Line 53: (LI et al., 2021). Misquoted edit
**Response:** Thank you to the reviewer for the careful review. The manuscript has been revised according to the review comments.

**Question 4:**The Introduction section lacks references to the latest relevant research, e.g. Wang et al., 2024. https://doi.org/10.1016/j.resconrec.2024.107898
**Response:** Thank you to the reviewer for the careful review. Based on the review comments of two reviewers, the introduction section of the manuscript was reorganized, and relevant literature from the past five years was cited to summarize the latest research progress in intercropping and multiple cropping modes. Please refer to the revised draft for details.

**Question 5:** Line 75: In wheat-maize intercropping system. You can refences the following research:https://doi.org/10.1016/j.catena.2023.107247

**Response:** Thank you to the reviewer for the careful review. According to the reviewer's comments, the research progress on intercropping patterns has been re searched, and discussions on research progress in optimizing fertilization and other aspects have been added. Please refer to lines 57-58 of the revised manuscript.

**Question 6:** Lines 79-80: A vague question. I recommend that authors clarify their research objectives, and to come up with a reasonable presupposition.
**Response:** Thank you to the reviewer for the careful review. In the last paragraph of the introduction, the research objectives, research plan, and survey content were added, as detailed in lines 87-90 of the revised manuscript. The basic assumptions of the study have been supplemented in the experimental design section (1.2), as shown in lines 116-119 of the revised manuscript.

**Question 7:** Line 87: This model can be used in many irrigation districts around the world, not just in experimental area.
**Response:** Thank you to the reviewer for the careful review. The diversified planting mode proposed in this study is based on the climatic conditions of the Yellow River irrigation area in Ningxia. Therefore, the resulting planting mode is suitable for the Yellow River irrigation area in Ningxia as well as other similar ecological regions worldwide. The last paragraph of the introduction has been revised; please refer to lines 94-96 of the revised draft for further details. The modified content is as follows:

“Suitable intercropping and multiple planting modes and crop species are proposed for the Yellow River Irrigation Area in Ningxia and other similar ecological regions worldwide, thus providing a scientific basis for the promotion of diversified planting modes.”

**Question 8:** Line 135-156: These formulas need to be supplied with a citation.
**Response:** Thank you to the reviewer for the careful review. The references have been supplemented, mainly referring to the following literature:

Zhang ZW, Li HY, Fu Q, Shi HB, Liu DP. Response Mechanism of Wheat /Maize Intercropping System under Different Water Stresses. Transactions of the Chinese Society for Agricultural Machinery. 2020; 51(1): 245-255.https://doi.org/10.6041/j.issn.1000-1298.2020.01.027

**Question 9:** Line 161: Intercropping

**Response:** Thank you to the reviewer for the careful review. Spelling error, corrected.

**Question 10:** Please replace all statistical units with the unit hectare (ha) instead of 667 m^2^, as this is not internationally common.

**Response:** Thank you to the reviewer for the careful review. The area unit mentioned in the manuscript has been revised to hectares according to the review comments.

**Question 11:**Table 2: Add line spacing or spaces so that each treatment is clearly distinguishable to the reader
**Response:** Thank you to the reviewer for the careful review. The treatments in Tables 2 and 3 have been separated by horizontal lines according to the review comments. Please refer to the revised manuscript for details.

**Question 12:** Line 212: Interspecies
**Response:** Thank you to the reviewer for the careful review. Spelling error, corrected.

**Question 13:**In fact, heat is the key determinant of polyculture. The authors were able to grow multiple crops in a single growing season because sufficient heat was available. Moreover, some crops are heat-loving crops and some are cool-loving. These differences in growth habits between crops lead to complementary ecological niches, which in turn determine yields. Please add relevant discussions：Chai et al., 2021, PNAS
**Response:** Thank you to the reviewer for the careful review. According to the review comments, a discussion on the impact of heat on intercropping patterns has been added, and the basis for selecting intercropping crops in this study has been introduced. The revised content can be found in lines 305-310.

**Question 13:** Duplicates appear in the references list, e.g. Wang 2022

**Response:** Thank you to the reviewer for the careful review. The references have been revised according to the journal format and duplicates have been removed.
